# The Development of New Agents for Post-Hematopoietic Stem Cell Transplantation Non-Infectious Complications in Children

**DOI:** 10.3390/jcm12062149

**Published:** 2023-03-09

**Authors:** Uri Ilan, Erica Brivio, Mattia Algeri, Adriana Balduzzi, Marta Gonzalez-Vincent, Franco Locatelli, Christian Michel Zwaan, Andre Baruchel, Caroline Lindemans, Francisco Bautista

**Affiliations:** 1 Princess Máxima Center for Pediatric Oncology, 3584 CS Utrecht, The Netherlands; 2 Department of Hematology/Oncology and Cell and Gene Therapy, Bambino Gesù Children Hospital, 00165 Rome, Italy; 3 Clinica Pediatrica Università degli Studi di Milano Bicocca, 20900 Monza, Italy; 4 Department of Stem Cell Transplantation, Hospital Infantil Universitario Nino Jesus, 28009 Madrid, Spain; 5 Department of Pediatric Hematology, AP-HP, Robert Debré Hospital, 75019 Paris, France; 6 Division of Pediatrics, University Medical Center Utrecht, 3584 CX Utrecht, The Netherlands; 7 Department of Stem Cell Transplantation, Regenerative Medicine Center, University Medical Center, 3584 CX Utrecht, The Netherlands

**Keywords:** new agents, post-transplant complications, clinical trial, children

## Abstract

Hematopoietic stem cell transplantation (HSCT) is often the only curative treatment option for patients suffering from various types of malignant diseases and some non-cancerous conditions. Nevertheless, it is associated with a high risk of complications leading to transplant-related mortality and long-term morbidity. An increasing number of therapeutic and prevention strategies have been developed over the last few years to tackle the complications arising in patients receiving an HSCT. These strategies have been mainly carried out in adults and some are now being translated into children. In this manuscript, we review the recent advancements in the development and implementation of treatment options for post-HSCT non-infectious complications in pediatric patients with leukemia and other non-malignant conditions, with a special attention on the new agents available within clinical trials. We focused on the following conditions: graft failure, prevention of relapse and early interventions after detection of minimal residual disease positivity following HSCT in acute lymphoblastic and myeloid leukemia, chronic graft versus host disease, non-infectious pulmonary complications, and complications of endothelial origin.

## 1. Introduction

Hematopoietic stem cell transplantation (HSCT) is an effective therapeutic option for a wide range of malignant and non-malignant disorders [1]. Despite its potentially curative effect, it is associated with a high risk of non-infectious complications that can virtually affect any organ and lead to transplant-related mortality and long-term morbidity [2,3]. These rates have progressively lowered due to improved supportive therapy, tailored conditioning regimens and better HLA typing, among others. Nevertheless, 2-year survival rates of approximately 65–75% with around 10% transplant-related mortality remains unacceptable [4,5]. Further improvements in outcomes following HSCT thus require new strategies to overcome the limitations of current approaches. Some have been developed over the last years, mainly in adults, and more recently in children [6,7,8].

In this review, we restricted ourselves to five prevalent categories of non-infectious post-HSCT complications in children: (a) graft failure (GF); (b) prevention of relapse (including interventions after detection of minimal residual disease (MRD) post-HSCT for acute lymphoblastic and myeloid leukemia (ALL, AML)); (c) systemic chronic graft versus host disease (cGVHD); (d) non-infectious pulmonary complications; and (e) complications of endothelial origin. We decided not to include studies focusing on acute GVHD since they have been recently reviewed [9]. Because these complications affect patients with both benign and malignant conditions after HSCT, we analyzed all studies regardless of the underlying disease.

We aim to provide an update on the clinical trial portfolio evaluating new strategies for post-HSCT non-infectious complications, and discuss potential future therapies and areas of improvement.

## 2. Materials and Methods

The website https://clinicaltrials.gov (Last accessed on 17 October 2022) was scrutinized to identify clinical trials and 17 searches were conducted with the following pre-defined terms: “Graft failure”, “Relapse” (restricted to “HSCT”), “Minimal residual disease”, “Stem Cell Transplant Complications”, “Transplant; Complication, Rejection”, “Chronic graft versus host disease” (cGVHD), “Bronchiolitis obliterans”, “Cryptogenetic organizing pneumonia”, “Idiopathic pneumonia syndrome”, “Diffuse alveolar hemorrhage”, “Veno-occlusive disease”, “Sinusoidal Obstruction Syndrome”, “Engraftment syndrome”, “Capillary Leak Syndrome”, “Thrombotic Microangiopathies”, “Thrombotic Thrombocytopenic Purpura”, and “Hemolytic Uremic Syndrome”.

Eligibility criteria were defined a priori. Only interventional phase I-III trials in children recipients of an HSCT between 1 January 2011 and 31 December 2021 were searched (last accessed 17 October 2022). Studies with unknown statuses were excluded. When the research did not yield pediatric studies, we enlarged it to the adult age interval to identify potential relevant studies for children.

PubMed was searched to cover the available literature on therapies for these complications using the four following MeSH terms: “Hematopoietic stem cell transplantation”, “children”, “complications”, and “clinical trial”.

## 3. Results

**Figure 1:**
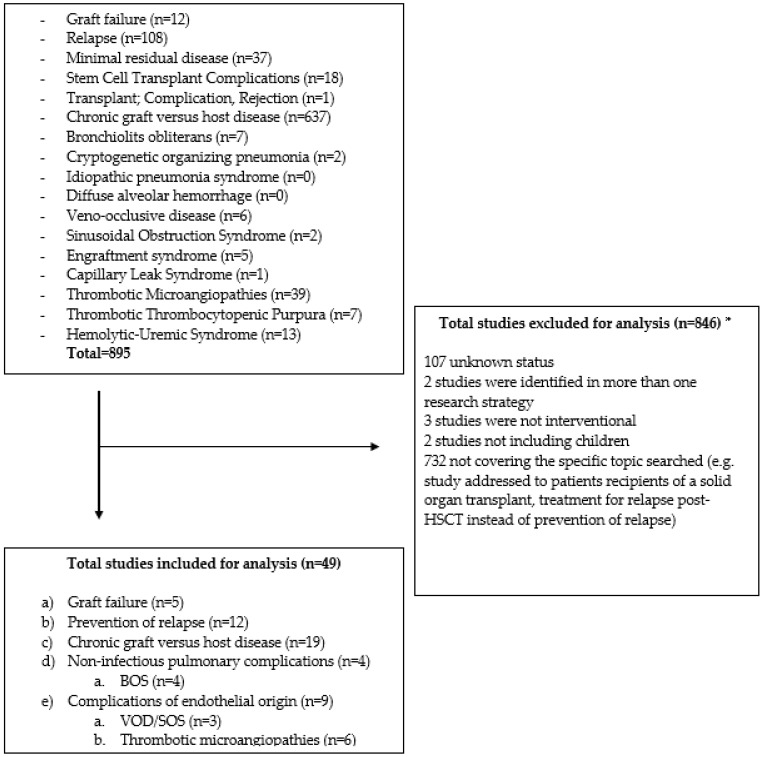
Flow diagram reporting the results of the search at clinicaltrials.gov (Last accessed 17 October 2022). BOS: bronchiolitis obliterans syndrome; HSCT: hematopoietic stem cell transplantation; SOS: sinusoidal obstruction syndrome; VOD: veno-occlusive disease. * Studies could be excluded for more than one reason, but they were counted only once in this figure.

**Figure 2:**
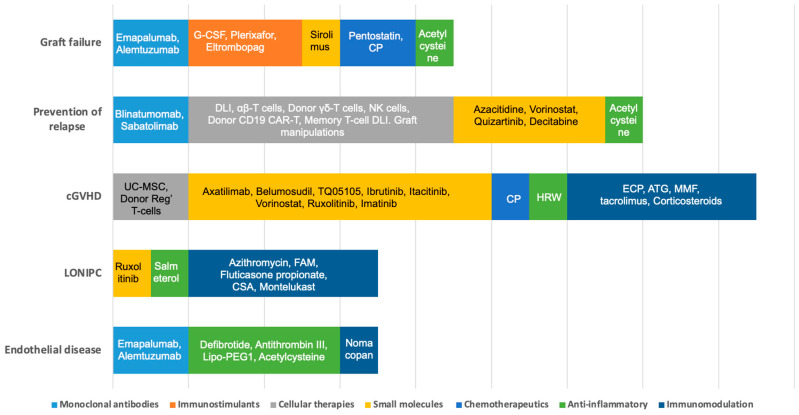
Strategies explored in children by mechanism of action. G-CSF: granulocyte colony-stimulating factor; CP: cyclophosphamide; DLI: donor lymphocyte infusions; NK: natural killer; CAR-T cells: chimeric antigen T cells; CIML-NK: cytokine-induced memory-like natural killer; cGVHD: chronic graft versus host disease; UC-MSC: umbilical cord mesenchymal stem cells; HRW: hydrogen-rich water; ATG: anti-thymocyte globulin; ECP: extracorporeal photopheresis; MMF: mycophenolate mofetil; FAM: fluticasone propionate, azithromycin, and montelukast sodium; CSA: cyclosporine A; TKI: tyrosine kinase inhibitor.

The search at clinicaltrials.gov yielded 895 studies (Figure 1). Forty-nine fulfilled the eligibility criteria: (a) graft failure (*n* = 5); (b) prevention of relapse (*n* = 12); (c) cGVHD (*n* = 19); (d) non-infectious pulmonary complications (*n* = 4); and (e) complications of endothelial origin (*n* = 9). Three additional studies for prevention of relapse were identified with cross-references (*) and one for GF (*) (Table 1). Figure 2 depicts the strategies explored in children by mechanism of action.

### 3.1. Graft Failure

Primary GF is characterized by the absence of initial donor cell engraftment; secondary GF occurs after initial engraftment and subsequent loss of donor cells not related to relapse, infection, or toxicity [1]. The incidence has been reported to be <5% in the matched allo-HSCT setting and 10% in haploidentical or cord blood transplant [2]. Well-established risk factors for GF are HLA mismatch, graft source, gender incompatibility between donor and receptor, non-malignant diseases, presence of anti-HLA antibodies, and splenomegaly in the receptor [1]. Treatment has mainly consisted of maximizing supportive care and tailoring immunosuppression, but in some cases the only alternative option is a second transplantation.

To reduce the recipient cellular activity, a phase 2 trial (NCT02105766) for patients with severe sickle cell disease and B-thalassemia receiving allogeneic peripheral blood stem cell (PBSC) transplants from HLA-matched family donors, is investigating whether low dose radiation (300 rads), oral cyclophosphamide, pentostatin, alemtuzumab, and sirolimus can reduce the engraftment failure rate. The intensified non-myeloablative approach aims to reduce the T cells mediating leukocyte rejection and the B/plasma cells that produce anti-donor erythrocyte antibodies. Another phase 2 study (NCT05126186) is testing the addition of post-transplant cyclophosphamide which targets alloreactive T cells after haploidentical HSCT for prevention of GF in patients with hematological diseases.

Plerixafor and G-CSF were evaluated for prevention of GF after TCR αβ-depleted grafts in patients with Wiskott--Aldrich syndrome (WAS). Plerixafor antagonizes the alpha chemokine receptor CXCR4, which plays an important role in stem cell homing. The combination with G-CSF mobilizes bone marrow stem cells into peripheral blood and blocks CXCR4 chemokine receptors to prevent stem cell homing, producing a free stromal space in the marrow for engraftment. Sixteen patients were prospectively included and compared with a historical cohort of eighteen patients. The conditioning regimen was treosulfan, fludarabine, and melphalan, with G-CSF 10 µg/kg on days −8, −7, −6, −5, and −4 and plerixafor 240 µg/kg on days −6, −5, and −4. The prospective and historical groups had comparable baseline characteristics. No patients in the prospective cohort developed GF, compared to 39% in controls [3]. Based on these findings, this combination is currently being tested in patients with chronic granulomatous disease, a non-malignant disease notorious for its risk of graft rejection (NCT03547830).

A phase 2 study (NCT04731298) has evaluated emapalumab, an interferon gamma (IFNγ)-blocking antibody, in children at high risk of GF. Serum levels of interferon (IFN)-γ and CXCL9 (a chemokine specifically induced by IFNγ) are found to be significantly higher in patients experiencing GF [5] but the results are not yet published.

Eltrombopag is being investigated in a phase 2 study (NCT03948529) to improve poor graft function after allo-HSCT. Eltrombopag interacts with the thrombopoietin receptor and stimulates proliferation and maturation of megakaryocytes. A randomized study in adults showed that eltrombopag leads to a higher percentage of patients achieving a platelet count of ≥50 × 10^9^/L compared to those given placebo [4].

N-acetylcysteine could be considered as a treatment for preventing graft failure as endothelial progenitor cells (EPCs) play a crucial role in the regulation of hematopoiesis and thrombopoiesis in the bone marrow microenvironment [7] and N-acetyl-L-cysteine (NAC, a reactive oxygen species [ROS] scavenger) can enhance bone marrow EPCs. In a prospective clinical trial (NCT03236220) including children, N-acetylcysteine was administered to patients with leukemia whose bone marrow endothelial cells were less than 0.1% detected before haploidentical hematopoietic stem cell transplantation and the primary endpoint was the incidence of poor graft function and prolonged isolated thrombocytopenia. They showed that N-acetylcysteine intervention was safe and effective in preventing the occurrence of PGF and PT in EC < 0.1% patients by promoting the dynamic reconstitution of BM ECs and CD34^+^ cells [7].

The effect of mesenchymal stem cells (MSCs) alone and combined with cord blood (CB) for GF have been tested post-autologous HSCT (including patients from 14 years old); the use of ex vivo-expanded MSCs were effective and did not result in GVHD or increase the risk of tumor relapse and the addition of CB had a superior effect on neutrophil reconstruction [8].

In conclusion, as the main risk factors and attributed biology mechanism are immune related [6], most of the studies concentrate on immune modulation to achieve better engraftment, by reducing recipient T cell activity, increasing graft mobilization by using GCSF, or increasing bone marrow proliferation with eltrombopag.

### 3.2. Prevention of Relapse

Recurrence of leukemia remains the leading cause for failure after allo-transplantation. The graft-versus-leukemia (GVL) effect can help preventing relapse and can be enhanced with early immune suppression withdrawal or cellular therapies [9]. Pharmacologic agents can also play a role at conditioning but mainly in post-HSCT maintenance after engraftment. A major question remains what is the best timing to start maintenance without inducing excessive toxicities (e.g., GVHD), compromising graft integrity, or interfering with other pharmacologic agents [10].

#### 3.2.1. Cellular Therapies

Donor lymphocyte infusion (DLI) is a minimally manipulated product that stimulates GVL reactions. However, the utility of DLI is limited by the risk of GVHD and by variable responses [11]. To enhance the response to DLI in preventing leukemia, the phase 2 NCT02458235 trial tested post-transplant consolidation with DLI and the hypomethylating agent (HMA) azacytidine. No severe toxicity attributable to azacytidine was observed and the combined intervention was deemed feasible and only 2 of the 11 patients developed a relapse [12]. In the phase 2 (NCT02673008) trial, preemptive DLI was administered monthly to patients with persistent MRD positivity after transplant and prophylactically to those when donor lymphocytes were available without active GHVD [13]. The combined approach of sequential intensified conditioning and DLI resulted in a reduced incidence of relapse compared to historical controls in patients with relapsed ALL and AML.

Tumor-reactive lymphocytes are being tested in clinical trials including αβ-T lymphocytes, γδ-T lymphocytes, natural killer (NK) cells, and NK-T cells. A clinical trial (NCT04439721) is testing the safety and effectiveness of a donor γδT cell infusion, a population of cells with both innate and adaptive immune properties with potent in vitro anti-tumor responses.

To increase the GVL effect without causing any GVHD, a phase 2 trial (NCT03849651) is being conducted where TCRαβ- and CD19-depleted graft infusions are followed by CD45RA-depleted DLI at least two weeks after engraftment. In addition, in this study for CD19-positive B-ALL patients, blinatumomab will be given at least one week post-DLI for 28 days.

In the allogeneic setting, donor NK cells can kill recipient cells through missing self-recognition and offer the advantage of inducing a GVL effect without necessarily promoting GVHD. The NCT04024761 trial is studying cytokine-induced memory-like natural killer (CIML-NK) cells plus IL-2 which leads to activation and expansion of the NK cells [14] in patients above 12 years with myeloid malignancies who relapsed or remain MRD positive after haploidentical HSCT.

The NCT02050347 trial tests donor CD19/CD28 chimeric receptor T cells in leukemia and lymphoma patients with residual disease at transplantation or post-transplant evidence of MRD positivity (or relapse).

#### 3.2.2. Hypomethylating Agents (HMA)

HMA may permit epigenetic manipulation of the alloreactive response, through conversion of alloreactive donor T cells into suppressive regulatory T cells via hypomethylation of the FOXP3 promoter and suppression of alloreactive T cell proliferation [15]. In a recent trial in adults with AML and myelodysplastic syndrome (MDS) who developed MRD positivity after complete remission after chemotherapy or HSCT, the participants were offered azacytidine; relapse free survival at 12 months was 58% [16]. On the other hand, a study of post-HSCT azacytidine maintenance in adults with AML and MDS, starting 40 to 100 days after allo-SCT and continuing for up to 12 months, failed to show relapse or survival benefits [17]. The NCT01995578 trial, also including children from 12 years old, tests azacytidine post-T cell-depleted allo-SCT for patients with MDS and AML. NCT04945096 will study decitabine maintenance to reduce relapse and restore the bone marrow microenvironment by repairing endothelial cells and endothelial progenitor cells, as well as cytokines and chemokines, which are crucial to hematopoietic cell proliferation and differentiation promoting platelet recovery. Co-administration of histone deacetylase inhibitors, such as vorinostat, is reported to improve the clinical activity of azacitidine in children with relapsed AML [18]. Based on this premise, the NCT03843528 trial is testing the addition of vorinostat to azacitidine as a post-HSCT maintenance treatment.

#### 3.2.3. Targeted Therapy

##### Tyrosine Kinase Inhibition

Multiple adult studies have reported that BCR-ABL TKI after HSCT improves relapse-free survival in patients with Philadelphia-positive ALL (Ph+ ALL) [19]. The only randomized trial published to date compared prophylactic (in MRD-negative patients) with preemptive (MRD-triggered) administration of imatinib in adult Ph+ ALL patients and demonstrated that both arms had improved survival when compared to historical controls, although most patients had to interrupt treatment due to poor tolerability. The NCT03793478 trial [20] is evaluating the administration of quizartinib, a potent and selective FLT3 TKI after transplantation in patients with FLT3-mutated AML.

##### Monoclonal Antibodies/Bispecific T-Cell Engagers

For BCP-ALL, five different studies are exploring blinatumomab’s effect on post-transplant patients (NCT03849651 was described in Section 3.2.1). A single arm prospective trial in adults with B-ALL evaluated the administration of four cycles of blinatumomab after HSCT at count recovery and at 6, 9, and 12 months. No differences in efficacy were seen when compared to a contemporary cohort [21]. In children, a phase 2 study (NCT04785547) will treat post-HSCT patients with 28 consecutive days of blinatumomab in two different cohorts, MRD-positive pre-HSCT and patients who became MRD positive after HSCT. The NCT02790515 trial is assessing the safety and feasibility of blinatumomab in the early post-engraftment period in patients with CD19+ malignancies after TCRαβ- and CD45RA-depleted haploidentical donor progenitor cell HSCT following a reduced intensity conditioning regimen. A similar approach is being used in NCT04746209, in which blinatumomab is administered at day +100 while administering αβ T cell- and B cell-depleted allogeneic hematopoietic cell transplantation. The NCT04044560 trial terminated earlier due to slow recruitment and explored blinatumomab given for up to four cycles.

For AML, gemtuzumab ozogamicin, an antibody against CD33 conjugated to the chemotherapy calicheamicin, has been evaluated as a maintenance treatment with azacitidine after HSCT in a small series of 10 AML adult patients. Concerns about this approach are related to hematologic toxicity and the risk of post-HSCT development of hepatic veno-occlusive disease (VOD) [22]. In addition, in a phase 1/2 study, sabatolimab, a humanized monoclonal antibody (hIgG4, S228P) directed against the human T-cell immunoglobulin domain and mucin domain-3 (TIM-3) aimed to enhance T-cell killing and inflammatory cytokine production by dendritic cells [23], will be combined with azacytidine to treat MRD-positive AML patients post-HSCT (NCT04623216).

### 3.3. Chronic Graft vs. Host Disease (cGVHD)

Chronic GVHD accounts for 25% of late non-relapse morbidity and mortality following allo-HSCT while also having a huge impact on patients’ quality of life [24]. cGVHD biology is characterized by hampered immune tolerance mechanisms where donor-derived T and B cells have an effect [16]. In addition, mechanisms of chronic inflammation result in fibrosis and scar formation [17]. Age also plays a role as thymic function and hormones can affect the immune reconstitution post-HSCT. From the recipient perspective, the most dominant risk factor is previous cGVHD and its severity. From the donor perspective, the most dominant risk factors are unrelated/mismatch donor, donor age, and female donor as well as peripheral blood source. In addition, conditioning with total body irradiation (TBI) also plays a role [18]. The long-term mortality is over 50% in cases of severe cGVHD [24].

Current first line standard of care is based on cGVHD severity, and includes steroids and calcineurin inhibitors (CNIs). Additionally, rituximab has been shown to be effective in attenuating the increased risk of infections and prolonged B-cell recovery [25].

#### 3.3.1. Cellular Therapies

Several clinical trials are focused on cellular therapies such as mesenchymal stem cells (MSCs) or manipulated donor T cells which have been shown to reduce incidence of GVHD. The phase 1/2 study, NCT05152160 [26,27] is investigating MSCs taken from umbilical cords (UCBs) as it has been shown that both donor bone marrow- and umbilical cord-derived MSCs have immunosuppressive properties. Another approach is donor manipulated T cells. NCT03683498 and NCT05095649 are using enriched CD25hi T cells (Tregs) from CD8 and/or CD19 pre-depleted leukapheresis products, which have been shown to be an effective and safe way to prevent GVHD [28] for patients who did not achieve remission after ruxolitinib.

#### 3.3.2. Targeted Therapies

Ibrutinib inhibits Bruton’s tyrosine kinase (BTK) and interleukin-2-inducible T-cell kinases (ITK) which regulate B-cell survival and activation of T cell subsets that drive immune reactivity towards health tissues [29]. Ibrutinib is approved for the second-line treatment of cGVHD in adults and recently also for children above 1 year of age with cGVHD after failure of one or more lines of systemic therapy, based on the results of the iMAGINE (NCT03790332) and iNTEGRATE trials (NCT02959944) [30]. JAK1/2 signaling is involved in many steps leading to inflammation, T-cell activation, and tissue damage in GVHD [31]. Ruxolitinib, a JAK1/2 inhibitor reduces the number of circulating activated T cells in patients with cGVHD [32], was prospectively evaluated in a phase III randomized study including children who had glucocorticoid-refractory acute GVHD after HSCT [33]. A total of 309 patients were included; both the response rate at 28 days and at day 56 were higher in the ruxolitinib group, as well as median free survival and overall survival. These results led to the approval of ruxolitinib for the treatment of steroid refractory cGHVD in children above 12 years of age. The NCT05121142 trial is investigating systemic ruxolitinib in children under 12 years of age with acute and chronic GVHD and the NCT03774082 in children under 18 years. TQ05105, another JAK2 inhibitor, is also being evaluate in this population (NCT04944043). Itacitinib, a JAK1 inhibitor, is being investigated in combination with cyclophosphamide and tacrolimus to prevent GVHD (NCT05364762). Belumosudil, a selective ROCK2 inhibitor which reduces follicular T helper cells by downregulating STAT3 and upregulating STAT5, has been approved for patients above 12 years of age with cGVHD after failure of at least two prior lines of treatment, based on the results of the phase II randomized study (NCT03640481) comparing two different doses (200 mg OD or 200 mg BID) [34]. The study remains open for patients under 18 years of age and another study is also evaluating belumosudil in patients above 12 years (NCT05567406). Axatilimab (SNDX-6352) is a humanized, full-length IgG4 antibody with high affinity to CSF-1R. Axalitamab affects the migration, proliferation, differentiation, and survival of monocytes and macrophages by binding to CSF-1R and blocking its activation by its two known ligands, CSF-1 and IL-34, and may be a novel therapeutic option for treatment of patients with GVHD and has shown preliminary efficacy in adult patients [35]. Two studies (NCT03604692, NCT04710576) are evaluating axalitamab in patients cGVHD after two or more lines of therapy. Vorinostat, an HDAC inhibitor which affects the balance of circulating T-cell subsets by increasing the regulatory T cells and decreasing Th1 and Th17 cells [36], will be investigated in a phase I/II clinical trial (NCT03842696) administered at three different doses. Imatinib, a tyrosine kinase inhibitor which inhibits both PDGF (platelet-derived growth factor) and TGF-β (transforming growth factor-β) intracellular signaling, was investigated in combination with mycophenolate mofetil (MMF) (NCT01898377); however, the study was terminated due to the introduction of a new treatment option.

#### 3.3.3. Chemotherapeutics

A phase 2 study will randomize patient to receive post-transplant cyclophosphamide in addition to anti-thymocyte globuline (ATG) given during the conditioning regimen (NCT04202835). The administration of high-dose cyclophosphamide post-HSCT in combination with MMF and tacrolimus will be evaluated in the NCT03128359 trial in patients receiving three different conditioning regiments including fludarabine combined with either melphalan, busulfan, or total body irradiation (TBI).

#### 3.3.4. Other Therapies

Additional experimental therapies to treat cGVHD are being investigated. NCT03083574 is investigating the impact and safety of extracorporeal photopheresis, which might affect the patient’s dendritic cells by changing the antigen-specific T-cell immunity and T-cell tolerance [37] after HSCT. In addition, hydrogen-rich water consumption, an FDA-acknowledged food additive with reactive oxygen species scavenging properties is being investigated in cGVHD patients in the NCT02918188 study.

### 3.4. Non-Infectious Pulmonary Complications

Lung injury occurs in up to one third of patients undergoing HSCT and carries significant risks of morbidity and mortality [38]. There are several factors which can contribute to the development of lung injury such as pre-existing lung disease, treatments prior to HSCT, and the co-existence GVHD.

#### 3.4.1. Bronchiolitis Obliterans Syndrome (BOS)

BOS is an obstructive disease resulting from collagen deposition and fibrosis in the peribronchiolar space [39] and shares the same etiological origin as cGVHD [16]. Treatment traditionally consists of supportive measures and immunosuppressive agents. Systemic immunosuppression with prednisone with or without other immunosuppressive treatments (i.e., cyclosporine, azathioprine) have generally resulted in transient or unsatisfactory effects. Anti-TNF treatment (i.e., etanercept and infliximab) to inhibit the tumor necrosis factor receptor and diminish the ensuing inflammation [40], have been tested and shown some improvement [41]. The beneficial effects of macrolides relate primarily to their anti-inflammatory and immunomodulation properties [42]. A meta-analysis of four studies including 90 patients receiving allogenic HSCT with BOS showed that azithromycin increased FEV1 by 30 mL, but with limited clinical impact; there is also evidence that azithromycin can result in worse airflow decline-free survival [43]. Other options have been investigated, considering the same etiological origin as cGVHD [44]. Second-line options include extracorporeal photopheresis and JAK inhibitors, such as ruxolitinib [45]. To date, published data are limited to small case series or case studies in adults or children with steroid-resistant BOS [46]. Similarly, the Bruton’s TKI ibrutinib may have a potential role, although no prospective data have been published thus far in this condition [29]. Belumosudil demonstrated activity in adults with cGVHD, a third of which had lung involvement [34] and may also be a promising agent for this complication. TKIs have also been studied in cGVHD considering their antifibrotic and anti-inflammatory properties, as inhibitors of both PDGF-R and TGFβ pathways and immunomodulatory effects, and being involved in both T-cell and B-cell responses [47]. Only limited evidence about its use and efficacy is available in children [48].

Local treatment with inhaled budesonide/formoterol improves FEV1 in patients with mild/severe BOS [49]. The combination with inhaled fluticasone, azithromycin, and montelukast (also known as FAM therapy) was tested in a phase 2 trial for adults and children (NCT01307462) and was reported to halt pulmonary decline and permit reductions in systemic steroid exposure [50]. A phase 2 study (NCT01287078) tested inhaled cyclosporine in 20 patients but only 11 completed the treatments through week 18 due to cough and bronchospasm. Inhaled cyclosporine led to an improvement or stabilization of pulmonary function and/or a decrease in systemic immunosuppression in 9 of 11 patients [51]. The ongoing NCT04908735 is testing whether the addition of ruxolitinib to early treatment with fluticasone/montelukast and steroids can reverse lung injury and reduce the frequency of BO. A randomized phase 3 trial (NCT04655508) is evaluating the efficacy of fluticasone and salmeterol compared to placebo on respiratory function in children above six years who underwent allo-HSCT transplantation with a decline of FEV1 ≥ 10% from pre-transplantation.

#### 3.4.2. Cryptogenetic Organizing Pneumonia (COP)

COP is associated with restrictive pulmonary dysfunction and, in contrast to BOS, its etiology is not strictly related to GVHD and considered as a clinico-pathologic entity of unknown etiology [52]. COP shows a response to steroids in more than 80% of cases but relapses are common when tapering steroids [39]. Steroids, azithromycin, montelukast, and symbicort (SAMS) therapy was tested in adults, but the trial (NCT01432080) was terminated early due to insufficient accrual. Bardoxolone, a synthetic triterpenoid, is being evaluating in adults (NCT02036970) with pulmonary hypertension (including patients with COP) due to its suppression of pro-inflammatory mediators, enhancement of nitric oxide bioavailability, and suppression of vascular proliferation, leading to prevention of tissue remodeling. No clinical trials were identified in children.

#### 3.4.3. Idiopathic Pneumonia Syndrome (IPS)

Lung injury in IPS occurs through two pathways, the TNF-α/LPS-dependent and IL6/IL17-dependent [53]. Treatment is based on corticoids. Anti-TNF therapies have been tested; the randomized study of etanercept and steroids vs. placebo showed no difference in response in adults, although the study was terminated early due to low accrual. In a phase II study in children, the complete remission rate was 71% and the 1-year survival was 63% [41]. Other agents have been addressed to the IL6/IL17 pathway, mainly tocilizumab and brodalumab [54]. No clinical trials were identified in children in clinicaltrials.gov.

#### 3.4.4. Diffuse Alveolar Hemorrhage (DAH)

DAH is occurs in 2–14% of recipients as a consequence of damage to the alveolar capillary membrane [39]. Treatment generally consists of high doses of methylprednisolone or low doses of steroids and aminocaproic acid, but the overall response to this treatment is disappointing [55]. No clinical trials were identified in children.

### 3.5. Complications of Endothelial Origin

#### 3.5.1. Veno-Occlusive Disease (VOD) or Sinusoidal Obstruction Syndrome (SOS)

VOD or SOS affects both sinusoidal endothelial cells and hepatocytes. In children, the incidence is higher than in adults, with an average incidence of 20% and can progress to multi-organ failure in 30–60% of cases [56]. This complication is due to the accumulation of toxic metabolites in patients with a reduced glutathione (GSH) enzymatic system that leads to endothelial injury. Patients with certain conditions such as neuroblastoma or osteopetrosis and recipients of an allo-transplant and prior exposure to inotuzumab or gemtuzumab, are at an increased risk of developing this complication [57]. To date, several agents have been evaluated mainly in adults including steroids, heparin, ursoedeoxycholic acid, and glutamine [58].

Limited data exist regarding the use of these agents in the context of controlled prospective trials in children. A phase 2 study including 50 pediatric patients evaluated a continuous heparin infusion (100 units/kg/day) from the first day of the preparative regimen to day +30 or until discharge compared to a historical control (*n* = 70) [59]. This study showed that heparin could be safely used in prophylaxis in children undergoing myeloablative therapy and transplantation, leading to a significantly lower incidence of moderate and serious VOD.

A phase 3 randomized trial in children showed that prophylactic defibrotide, a pro-fibrinolytic agent, reduced the incidence of day +30 post-HSCT VOD by 8% [60]. It is the only agent approved by the FDA and EMA to treat pediatric severe VOD and it is associated with higher rates of survival at day +100 compared to historical controls (38.2 vs. 25%) when defibrotide was used in patients with established VOD/SOS [61]. A randomized phase 3 study in children and adults with high risk for developing VOD, compared the efficacy of prophylactic defibrotide vs. best supportive care (NCT02851407). In a preliminary report, 372 patients were included; no significant differences were found between both groups regarding the VOD/SOS-free survival by day 30 (85% vs. 80%) [62].

Pre-clinical studies have shown that antithrombin III (ATIII) has anti-inflammatory properties by binding to endothelial membranes and increasing prostacyclin synthesis, limiting platelet aggregation, and decreasing pro-inflammatory cytokine production. To date, studies investigating prophylactic or therapeutic ATIII have produced contradictory results [63]. A phase 2 study has evaluated the ATIII effect on children and adults with hepatic VOD post-HSCT (NCT01886248), but results are not yet available.

The use of prostaglandin E1 (PGE1) in prophylaxis has shown to decrease the incidence of VOD in adults and children due to its anti-inflammatory properties [64,65]. Based on this premise, the NCT02338440 study has investigated its pharmacokinetic properties and the incidence of VOD after prophylactic lipo-PGE1, but the results are not yet available.

#### 3.5.2. Engraftment Syndrome (ES)

ES is defined as the appearance of different clinical manifestations when the first neutrophils in the peripheral blood indicate the engraftment [66]. ES is the result of a systemic endothelial damage by the release of pro-inflammatory cytokines (IL-2, TNF-α, IFN-γ, IL-6) and products of degranulation and oxidative metabolism from neutrophils. Although the suggested therapy consists of corticosteroid therapy, controlled trials have not been performed to assess its benefit. A trial in adults is testing the prophylactic budesonide from day 5 in autologous and allogeneic stem cell transplant recipients (NCT05509933). Other approaches are cytokine targeted therapies such as tocilizumab and anakinra. In a single arm study, cord blood recipients of any age with steroid refractory ES, received tocilizumab at 4–8 mg/kg. Clinical responses with a decrease in body temperature and disappearance of skin rash was observed in all patients (*n* = 11, age 2–22) [67]. No clinical trials were identified in children.

#### 3.5.3. Capillary Leak Syndrome (CLS)

CLS consists of fluid loss into the interstitial spaces, secondary to capillary damage after endothelial injury. The incidence in children is 5% and correlates with infections, higher incidence of other endothelial complication, and GVHD [68]. Therapy is limited to supportive care. Despite the identification of plausible biological markers like the vascular endothelial growth factor (VEGF), an inducer of vascular permeability, targeted therapy is not available and reports of anti-VEGF therapy are anecdotal [69]. No clinical trials were identified in children.

#### 3.5.4. Transplant-Associated Thrombotic Microangiopathies (TA-TMAs)

TA-TMAs are characterized by microangiopathic hemolytic anemia and thrombocytopenia due to platelet clumping and mechanical red blood cell disruption in the microcirculation [70] which is associated with increased mortality and related to the use of calcineurin inhibitors [71]. The complement system is activated through the classical and alternative pathways producing deposits of C4d or C5b-9 fractions, respectively [72]. Defibrotide has been tested in a phase 2 study where it was administered at 6.25 mg/kg IV 6h prior to the start of conditioning until day +21 [73]. Twenty-five children were included and 4% developed TA-TMA compared to 18–40% in historical controls, suggesting preliminary evidence of activity in this setting. N-acetylcysteine was prospectively evaluated in a randomized phase 3 study including children (NCT03252925). A total of 140 patients were included (13% under 18 years of age). The incidence of TA-TMA at 100 days after HSCT was lower in the intervention group compared to the placebo group (7.6% vs. 19.5%).

The complement blocker eculizumab, used in the treatment of atypical hemolytic uremic syndrome (HUS), appears to be an effective therapy for TA-TMA in children; a retrospective study showed that the use of eculizumab resulted in a significant improvement in overall survival compared to historical controls (66% vs. 16%) [74]. The NCT03518203 trial tested eculizumab in pediatric and adult patients with high risk of developing TA-TMA, but results are not yet known. Ravulizumab is a humanized monoclonal antibody that inhibits complement protein C5, with the same indication as eculizumab for HUS but with a longer half-life [75]. Two trials are testing Ravalizumab in patients with TA-TMA including children (NCT04557735, NCT04543591). Nomacopan inhibits terminal complement activation by binding to C5 and preventing its cleavage and activation. A trial (NCT04784455) is currently ongoing in children. Narsoplimab, an inhibitor of mannan-binding lectin-associated serine protease-2 in the alternative complement activating pathway, showed efficacy in the treatment of adult patients with TA-TMA by improvement of laboratory markers and organ function [76], leading to the approval in the adult population. An expanded access program is available in children (NCT04247906) and a pediatric trial of narsoplimab for TA-TMA is beginning soon.

## 4. Discussion

We have provided a comprehensive overview of the current landscape of the standard of care and clinical trials evaluating new interventions for children who experience non-infectious complications following HSCT.

Although several new treatment options have emerged from the adult post-transplant setting there is a clear paucity of ongoing clinical trials in the pediatric population. Transplant procedures and the incidence of complications are different in children and results obtained from adult studies can therefore not always be easily translated to children.

We only identified five active trials addressing the prevention or treatment of graft failure in pediatric patients (Table 1). This is probably related to the specific pediatric non-malignant indications for HSCT at a young age for inborn error conditions, which share multiple risk factors for graft failure, such as pre-transplant patient’s characteristics (i.e., extensive marrow fibrosis, transfusion history, splenomegaly) and conditioning regimens different from those for malignant diseases [77,78]. Salvage treatment with a second transplant, preceded by a highly immunosuppressive regimen, is frequently employed for rescue, but still the associated toxicity remains high and outcomes are poor [79]. Therefore, new strategies are needed to prevent or revert this situation. Most of them are now focused on exposure targeted conditioning, and modifying the immune context and bone marrow microenvironment.

In malignant diseases, the prevention of relapse and GVHD management post-transplant remain the most important factors to improve the post-HSCT outcome. For prevention of relapse, there are a wide range of potential treatments available considering all the new targeting agents and non-chemotherapy approaches tested pre-HSCT. Nevertheless, the specificity of the post-HSCT setting needs to be carefully evaluated to reach solid conclusions. The main concern is the timing the start of anti-cancer agents after HSCT to avoid damage to the new graft or increased toxicity, but still early enough for preventing relapse. Current evidence suggests that maintenance therapy in AML is safe and associated with improved survival in adults but the pediatric field is lagging behind these experiences, both in the pre- and post-transplant setting [80]. This is partially due to a general delay in development of new agents in children, justified by safety reasons, with pediatric trials starting only after the first results in adults, and additionally due to the rarity of the pediatric patients, especially transplanted patients [81,82]. Improvement would require the effort of large and international clinical trials, as for instance the EsPhALL2010 (NCT00287105) trial, which recommended the use of imatinib post-HSCT for Ph+ ALL or the use of FLT3 inhibitors post-HSCT in AML patients [83].

Nevertheless, the compliance to the study recommendations is not yet known as the results are not yet available. BiTE approaches Other than blinatumomab and cell therapies have not reached the post-transplant setting. In the cGVHD setting, the examples from ibrutinib, ruxolitinib, and belumosudil that have been evaluated in parallel in adults and children within the scope of registration studies and led to regulatory approval for its use in children are definitely a way forward to accelerate the development of new drugs in children.

We could only identify a few trials in children for the non-infectious pulmonary complications and endothelial complications. These complications often share mechanisms of etiology (GVHD and BOS or VOD/SOS and TA-TMA). In addition, interaction between other complications and simultaneous appearance of these pulmonary and endothelial complications, such as endothelial damage coexisting and possibly fueling GVHD, can together potentially affect all organ functions and should be considered when a new treatment approach is tested. This also highlights the important of advancing therapeutic interventions for endothelial complications in children. Often small case series report the efficacy of an approach in pediatric patients, such as for eculizumab for TA-TMA or different treatment options for BOS, but systematic clinical trials are missing and therefore there is no pediatric label for most of the possible treatments for these conditions [74]. The recent approval of narsoplimab in adults with TA-TMA, may lead soon to the opening of a pediatric study in this condition. An exception is represented by the VOD treatment with defibrotide, which achieved a pediatric label thanks to the results of the clinical trials including pediatric patients.

We acknowledge the limitation of having missed studies that were posted in other databases different from clinicaltrials.gov. We nevertheless performed an exhaustive scrutiny of studies using the advance search including multiple and overlapping terms. We also looked for cross-references where available and performed an additional search in PubMed to minimize the possibility of missing relevant studies.

In general, we have observed a paucity of clinical trials for many different conditions, such as COP, capillary leak syndrome or engraftment syndrome, and in a larger setting of post-HSCT complications. Therefore, there is an urgent need to perform such studies to improve the outcome after HSCT, which is the only curative option for a wide range of malignant and non-malignant disorders. This requires a concerted action among the involved stakeholders led by academics having the expertise and experience to prioritize the areas that require urgent improvements.

## Figures and Tables

**Table 1:** Ongoing clinical trials in children with post-hematopoietic stem cell transplantation non-infectious complications.

	Disease of Interest (If Specified)	Ages	Phase	Investigational Agent(s)	Intervention	Trial Number	Estimated Enrollment (Number of Participants)	Status
**Graft failure**								
	Sickle cell disease and β-thalassemia	4 Years and Older	Phase 2	Alemtuzumab, low dose radiation, oral cyclophosphamide, pentostatin, and sirolimus	N/A	NCT02105766	162	Active, recruiting
	All hematological diseases	3 Years to 70 Years	Phase 2	Cyclophosphamide (CP)	Post-transplant preventive CP at 50 mg/kg/day at D + 3 and D + 4	NCT05126186	35	Active, recruiting
	Chronic granulomatous disease	1 Month to 24 Years	Phase 2	Plerixafor/G-CSF	Both agents are administered before transplant	NCT03547830	17	Active, recruiting
	Non-malignant diseases, leukemia, and lymphoma	1 Year and Older	Phase 2	Emapalumab	Given IV from day 0 every 3–4 days for 15 doses or engraftment evidence at 6 mg/kg (first dose) and 3 mg/kg (subsequent doses)	NCT04731298	250	Terminated
	Leukemia and patients with poor graft function criteria	6 Years and Older	Phase 2	Eltrombopag	Eltrombopag OD at 50 mg/day starting not earlier than D60 post-HSCT. In the absence of response, dose can be increased up to 150 mg/day in blocks of 50 mg	NCT03948529	25	Active, recruiting
	Acute leukemia patients	15 Years to 60 Years	Phase 2	*N-acetyl-L-cysteine	N-acetyl-L-cysteine given orally at dosages of 400 mg three times per day from 14 days pre-allotransplant to 2 months after allotransplant	NCT03236220	35	Completed
**Prevention of relapse including early interventions after detection of MRD positivity following HSCT in ALL and AML**								
	**Cellular therapy**							
	ALL, AML	10 Years to 65 Years	Phase 1	Donor γδT cell infusion	One infusion of 0.5 × 10^6–8^ × 10^7^ γδT/kg	NCT04439721	5	Active, recruiting
	AML, MDS, JMML	1 Year and Older	Phase 1	Cytokine-induced memory-like natural killer (CIML-NK) cells	CIML-NK given IV on day 0 with IL-2, three doses of fludarabine from day −5 to −3 and two doses CP on day −4 and −4	NCT04024761	50	Active, recruiting
	CD19 positive B-ALL/B-CLL/NHL	All	Phase 1	CD19/CD28 CAR T cells derived from donor	Maximum of six doses, 4 to 6 weeks apart, of CAR-T	NCT02050347	40	Active, recruiting
	Leukemia, lymphoma, and myeloproliferative diseases	Up to 21 Years	Phase 2	* TCRαβ-depleted progenitor cell graft with additional memory T cell DLI and blinatumomab	CD45RA-depleted DLI two weeks after engraftment of TCRα/β+ and CD19+-depleted, for CD19 pos; Blinatumomab will be given at least one-week post-DLI	NCT03849651	140	Active, recruiting
	ALL, AML, JMML, MDS	Up to 29 Years	Phase 2	Azacitidine and DLI	Up to seven cycles of low dose azacitidine (40 mg/m^2^ IV/SC daily × 4 days) at six weekly intervals and additional cycles according to risk levels	NCT02458235	17	Completed
	Acute leukemia	14 Years to 65 Years	Phase 2/3	DLI	DLI administered at a median dose of 1.0 (range 0.7–1.4) × 10^8^ mononuclear cells/kg once by day +60 and further based on MRD and GVHD status	NCT02673008	206	Completed
	**Hypomethylating agents (HMA)**							
	AML, MDS, JMML, MPAL	1 Year to 21 Years	Phase 1	Vorinostat/azacitidine	Two cycles of standard post-transplant azacitidine followed by vorinostat orally	NCT03843528	15	Active, recruiting
	AML, MDS	1 Year to 75 Years	Phase 2	Low dose of azacitidine	Azacitidine at 32 mg/m^2^ SC for 5 days every 28 days. At 60–120 days post-T-cell depletion of allo-HSCT	NCT01995578	32	Active, not recruiting
	All hematological diseases	10 Years to 70 Years	Phase 3	Decitabine and acetylcysteine	Decitabine (20 mg/m^2^/d) on days −10 to −8 and acetylcysteine from day −10 to +365	NCT04945096	100	Active, not yet recruiting
	**Targeted therapy**							
	AML	12 Years to 99 Years	Phase 1/2	Sabatolimab	Sabatolimab will be given every 4 weeks in combination with azacitidine	NCT04623216	59	Active, recruiting
	AML FLT3 ITD	1 Month to 21 Years	Phase 1/2	* Quizartinib	Quizartinib once daily starting on day 6 and continuing through to day 28	NCT03793478	65	Active, recruiting
	B-ALL	Up to 25 Years	Phase 2	αβ T cell- and B cell-depleted allogeneic hematopoietic cell transplantation (HCT) followed by blinatumomab	Twenty-eight-day continuous infusion of blinatumomab starting on day 100 post-transplant	NCT04746209	25	Active, recruiting
	Leukemia, lymphoma and myeloproliferative diseases	Up to 21 Years	Phase 2	TCRαβ- and CD45RA-depleted haploidentical donor progenitor cell transplantation followed by post-HSCT Blinatumomab	Continuous IV blinatumomab infusion at least 2 weeks post-engraftment for CD19-positive patients	NCT02790515	52	Active, recruiting
	B-ALL	6 Months to 21 Years	Phase 2	* Blinatumomab	Blinatumomab over a 28-day cycle. MRD-positive patients before HSCT start between day +60–+100 and for patients, who become MRD positive post-HSCT between day +60–+360	NCT04785547	32	Active, recruiting
	B-ALL	1 Year and Older	Phase 2	Blinatumomab	Blinatumomab for 6 weeks (4 weeks followed by a 2-week treatment-free period) for up to four cycles	NCT04044560	8	Terminated
**Chronic GVHD**								
	**Cellular therapy**							
		All	Phase 1	Donor regulatory T cells	CD25hi regulatory T cells from CD8 and/or CD19 pre-depleted leukapheresis products will be given in three dose levels	NCT03683498	16	Completed
		14 Years to 70 Years	Phase 1/2	Umbilical cord mesenchymal stem cells	N/A	NCT05152160	10	Active, recruiting
		All	Phase 2	Donor regulatory T cells	2 × 10^6^ cells/kg dose of regulatory T cell-enriched infusion	NCT05095649	15	Active, recruiting
	**Targeted therapy**							
		Up to 18 Years	Phase 1	Ruxolitinib	Ruxolitinib BID daily	NCT05121142	28	Active, recruiting
		1 Year to 21 Years	Phase 1/2	Ibrutinib	Ibrutinib orally once daily	NCT03790332	59	Active, not recruiting
	ALL, AML, CML, MDS, mature B-cell malignancies	3 Years to 39 Years	Phase 1/2	Vorinostat	Vorinostat at 30, 45, or 60 mg/m^2^ BID orally from day −10 days, until day +30 post-transplant.Haploidentical BMT recipients: same intervention but starting from day +5	NCT03842696	49	Active, recruiting
		6 Years and Older	Phase 1/2	Axatilimab (SNDX-6352)	SNDX-6352 IV will be given at a dose of 0.15–3 mg/kg	NCT03604692	40	Active, not recruiting
		12 Years and Older	Phase1/2	TQ05105	TQ05105 10 mg given orally, twice daily in 28 day-cycle	NCT04944043	97	Active, recruiting
		28 Days to 18 Years	Phase 2	Ruxolitinib	Ruxolitinib 5 mg BID	NCT03774082	46	Active, not recruiting
	ALL, AML, CML, MDS, myelofibrosis	Up to 80 Years	Phase 2	Itacitinib	CP IV QD on days 3 and 4, itacitinib PO QD on days 5–100, and tacrolimus IV or PO on days 6–65	NCT05364762	50	Active, not yet recruiting
		12 Years and Older	Phase 2	Belumosudil	Belumosudil 200 mg once or twice daily according to randomization	NCT03640481	175	Active, recruiting
		12 Years and Older	Phase 2	Belumosudil	Belumosudil orally, OD, or BID (if taking CYP3A4 inhibitors or proton pump inhibitors)	NCT05567406	12	Active, not yet recruiting
		2 Years and Older	Phase 2	Axatilimab (SNDX- 6352)	Axatilimab 0.3–3 mg/kg IV every 2 weeks for up to 2 years	NCT04710576	210	Active, not recruiting
		Up to 21 Years	Phase 2	Mycophenolate mofetil (MMF) and imatinib	MMF 15–20 mg/kg BID and imatinib QD 260 mg/m^2^/d	NCT01898377	9	Terminated
		12 Years and Older	Phase 3	Ibrutinib in combination with corticosteroids	Ibrutinib 420 mg is given orally OD starting on day 1 until cGVHD progression; in addition, 1 mg/kg/d prednisone OD until unacceptable toxicity or until participant is successfully tapered from the prednisone	NCT02959944	193	Completed
	**Chemotherapeutics**							
	AML, MDS	16 Years to 70 Years	Phase 2	Cyclophosphamide	ATG, 4.5 mg/kg IV on days −2, −1, and +1 and CP at 50 mg/kg IV daily on days +3 and +4 vs. ATG alone	NCT04202835	80	Active, recruiting
	ALL, AML, CML, MDS, CLL, Lymphoma	5 Years to 75 Years	Phase 2	Tacrolimus, high dose cyclophosphamide, and MMF	CP on days 3–4, mycophenolate mofetil TID on day 5 and stopping on day 35 if no severe GVHD is present. Tacrolimus IV continuously on days 5–180 with a taper beginning on day 90 in the absence of disease progression or unacceptable toxicity	NCT03128359	38	Active, not recruiting
	**Other**							
		All	Phase 2	Extracorporeal photopheresis	Six cycles of extracorporeal photopheresis every 2 weeks	NCT03083574	100	Active, recruiting
		up to 65 Years	Phase 2	Hydrogen-rich water	Hydrogen-rich water 4 mL/kg orally TID one day	NCT02918188	21	Active, recruiting
**Noninfectious pulmonary complications**								
		5 Years to 25 Years	Phase 2	Ruxolitinib	Ruxolitinib orally twice daily for 24 weeks plus standard fluticasone/montelukast and steroids	NCT04908735	40	Active, recruiting
		6 Years to 99 Years	Phase 2	Fluticasone propionate, azithromycin, and montelukast sodium (FAM)	Fluticasone propionate inhaled PO BID, azithromycin PO 3 days a week, and montelukast sodium PO QD for 6 months	NCT01307462	36	Completed
		10 Years to 80 Years	Phase 2	Cyclosporine inhalation solution	Cyclosporine inhalation solution (CIS) 150 mg three times weekly during weeks 1–5. Dose escalated to 300 mg three times weekly from weeks 6–8 until week 19	NCT01287078	25	Completed
		6 Years to 17 Years	Phase 3	Fluticasone propionate and salmeterol	Inhaled fluticasone propionate 50 or 125 μg and 25 μg salmeterol, BID from randomization and until 6 months	NCT04655508	243	Active, recruiting
**Complications of endothelial origin**								
	**VOD/SOS**							
		**up to 65 Years**	Phase 2	Antithrombin-III	AT-III at units required (IU)/kg = 50 + [(desired-baseline AT-III level) × weight (kg)/1.4]	NCT01886248	32	Active, not recruiting
		**All**	Phase 2/3	Lipoprostaglandin E1	Dose of 1.5 mcg/kg/day, continuous infusion	NCT02338440	30	Active, not recruiting
		**1 Month and Older**	Phase 3	Defibrotide	Defibrotide 25 mg/kg/day IV in addition to best supportive care, day before the first day of the conditioning for a recommended minimum of 21 days	NCT02851407	372	Completed
	**TA-TMA**							
		**All**	Phase 2	Eculizumab	Eculizumab IV for 24 weeks	NCT03518203	23	Active, recruiting
		**up to 30 Years**	Phase 2	Defibrotide	Defibrotide 6.25 mg/kg administered intravenously for 28–35 days	NCT03384693	25	Completed
		**All**	Phase 3	N-Acetylcysteine	50 mg/kg orally	NCT03252925	170	Completed
		**12 Years and Older**	Phase 3	Ravulizumab	Weight-based doses of ravulizumab will be administered intravenously as loading dose regimen followed by maintenance dosing every 8 weeks plus best supportive care (BSC) vs. placebo	NCT04543591	184	Active, not recruiting
		**28 Days to 17 Years**	Phase 3	Ravulizumab	Weight-based doses of ravulizumab administered IV as a loading dose regimen followed by maintenance every 4 or 8 weeks plus best supportive care	NCT04557735	40	Active, recruiting
		**6 Months to 18 Years**	Phase 3	Nomacopan	NA	NCT04784455	50	Active, recruiting

* Studies identified with cross references. ALL: acute lymphoblastic leukemia; AML: acute myeloid leukemia; ATG: anti-thymocyte globulin; BID: bis in die: BSC: best supportive care; CP: cyclophosphamide; CAR-T cells: chimeric antigen T cells; CIML-NK: cytokine-induced memory-like natural killer; DLI: donor lymphocyte infusions; G-CSF: granulocyte colony-stimulating factor; GVHD: graft versus host disease; HSCT: hematopoietic stem cell transplantation; IV: intravenous; JMML: juvenile myelomonocytic leukemia; SOS: sinusoidal obstruction syndrome; TA-TMA: transplant-associated thrombotic microangiopathies; MDS: myelodysplastic syndrome; MM: mycophenolate mofetil; MRD: minimal residual disease; N/A: not available; OD: once a day; PO: pero os (orally); QD: four times a day; SC: sub-cutaneous; TID: three times a day; TKI: tyrosine kinase inhibitor; VOD: veno-occlusive disease.

## Data Availability

The data were provided in the manuscript.
